# Nanoparticle delivery of grape seed-derived proanthocyanidins to airway epithelial cells dampens oxidative stress and inflammation

**DOI:** 10.1186/s12967-018-1509-4

**Published:** 2018-05-23

**Authors:** S. Castellani, A. Trapani, A. Spagnoletta, L. di Toma, T. Magrone, S. Di Gioia, D. Mandracchia, G. Trapani, E. Jirillo, M. Conese

**Affiliations:** 1 Department of Medical and Surgical Sciences, University of Foggia, Foggia, Italy; 2 Department of Pharmacy-Drug Sciences, University of Bari “Aldo Moro”, Bari, Italy; 3 Laboratory “BioProducts and BioProcesses”, ENEA Research Centre Trisaia, Rotondella, MT Italy; 4 Department of Basic Medical Sciences, Neuroscience and Sensory Organs, University of Bari “Aldo Moro”, Bari, Italy

**Keywords:** Oxidative stress, NF-κB, Airway epithelial cells, Solid lipid nanoparticles, Uptake, Apoptosis, Necrosis, Proanthocyanidins

## Abstract

**Background:**

Chronic respiratory diseases, whose one of the hallmarks is oxidative stress, are still incurable and need novel therapeutic tools and pharmaceutical agents. The phenolic compounds contained in grape are endowed with well-recognized anti-oxidant, anti-inflammatory, anti-cancer, and anti-aging activities. Considering that natural anti-oxidants, such as proanthocyanidins, have poor water solubility and oral bioavailability, we have developed a drug delivery system based on solid lipid nanoparticles (SLN), apt to encapsulate grape seed extract (GSE), containing proanthocyanidins.

**Methods:**

Plain, 6-coumarin (6-Coum), DiR- and GSE-loaded SLN were produced with the melt-emulsion method. Physicochemical characterization of all prepared SLN was determined by photon correlation spectroscopy and laser Doppler anemometry. MTT assay (spectrophotometry) and propidium iodide (PI) assay (cytofluorimetry) were used to assess cell viability. Flow cytometry coupled with cell imaging was performed for assessing apoptosis and necrosis by Annexin V/7-AAD staining (plain SLE), cell internalization (6-Coum-SLN) and reactive oxygen species (ROS) production (SLN-GSE). NF-κB nuclear translocation was studied by immunofluorescence. In vivo bio-imaging was used to assess lung deposition and persistence of aerosolized DiR-loaded SLN.

**Results:**

Plain SLN were not cytotoxic when incubated with H441 airway epithelial cells, as judged by both PI and MTT assays as well as by apoptosis/necrosis evaluation. 6-Coum-loaded SLN were taken up by H441 cells in a dose-dependent fashion and persisted into cells at detectable levels up to 16 days. SLN were detected in mice lungs up to 6 days. SLN-GSE possessed 243 nm as mean diameter, were negatively charged, and stable in size at 37 °C in Simulated Lung Fluid up to 48 h and at 4 °C in double distilled water up to 2 months. The content of SLN in proanthocyanidins remained unvaried up to 2 months. GSE-loaded SLN determined a significant reduction in ROS production when added 24–72 h before the stimulation with hydrogen peroxide. Interestingly, while at 24 h free GSE determined a higher decrease of ROS production than SLN-GSE, the contrary was seen at 48 and 72 h. Similar results were observed for NF-κB nuclear translocation.

**Conclusions:**

SLN are a biocompatible drug delivery system for natural anti-oxidants obtained from grape seed in a model of oxidative stress in airway epithelial cells. They feature stability and long-term persistence inside cells where they release proanthocyanidins. These results could pave the way to novel anti-oxidant and anti-inflammatory therapies for chronic respiratory diseases.

**Electronic supplementary material:**

The online version of this article (10.1186/s12967-018-1509-4) contains supplementary material, which is available to authorized users.

## Background

Chronic respiratory diseases, such as allergic asthma, chronic obstructive pulmonary disease (COPD), idiopathic pulmonary fibrosis (IPF), and cystic fibrosis (CF) represent a significant cause of morbidity, the fourth leading cause of death in the Western world and a significant social burden to be afforded. In particular, allergic asthma is increasing in its prevalence [1], and mortality for COPD increased in the last 30–40 years [2]. Another chronic lung disease, whose incidence is increasing due to the progress in diagnostics and therapeutics is CF [3]. The total cost of respiratory diseases in Europe is more than €100 billion per year. In general, the interaction of host genetic factors with the environment generate the pathologic triad in chronic respiratory diseases: persistent inflammation, protease–anti-protease imbalance, and oxidative stress. Oxidative stress is generated mainly by resident epithelial cells and macrophages, as well as by infiltrating inflammatory cells, which are represented by polymorphonuclear cells [4–6]. Initiating factors, including allergen exposure (asthma), cigarette smoking (COPD), defects in the CFTR in CF, and bacterial infection (COPD and CF), set up a vicious cycle of inflammation, oxidative stress and mucus overproduction.

Pulmonary drug delivery is widely accepted as the optimal approach for the first-line therapy against asthma and COPD. In addition, there are other diseases of notable social interest, as CF, tuberculosis and lung cancer, that can be treated with locally-acting drugs by pulmonary delivery [7, 8]. The treatment of respiratory diseases by direct administration of locally-acting drugs to the lungs offers several advantages including a high drug concentration at the site of action, rapid onset, low concentration in the systemic circulation and, hence, reduced side effects.

Polyphenol compounds are enriched in grape seed and skin, including proanthocyanidins, anthocyanins, flavonols, flavanols, resveratrol and phenolic acids. All these compounds are endowed with well-recognized anti-oxidant, anti-inflammatory, anti-cancer, anti-microbial and anti-aging activities [9]. However, the major hurdles of dietary polyphenols include their poor water solubility and oral bioavailability [10]. Thus, novel delivery systems are needed to address these problems. In the present study, we wished to evaluate if this objective can be achieved by using solid lipid nanoparticles (SLN) [11]. Besides control and/or target drug release, SLN are endowed with improved stability of pharmaceuticals, high and enhanced drug content as compared to other carriers, excellent biocompatibility since most lipids are biodegradable, they avoid organic solvents, and are more affordable, i.e. they are less expensive than polymeric/surfactant based carriers [12, 13]. However, the usefulness of SLN in mediating controlled and sustained delivery of polyphenol compounds as anti-oxidant and anti-inflammatory agents in the lung has not been explored yet.

In this study, we propose to vehicle natural substances obtainable from the red grape seed (polyphenols), widely produced in Apulia Region, via SLN into airway epithelial cells with the aim to reduce oxidative stress and inflammation.

## Methods

### Materials

Gelucire^®^ 50/13 was kindly donated by Gattefossè (Milan, Italy). Grape seed extract (GSE), containing 95.0–105.0% of proanthocyanidins was kindly provided by Farmalabor (Canosa di Puglia, Italy). Tween 85, methanol absolute grade, chloroform, sodium carbonate anhydrous, Folin–Ciocalteau reagent were purchased from Sigma Aldrich (Milan, Italy). DiR (1,1′-dioctadecyl-3,3,3′,3′-tetramethylindotricarbocyanine iodide) was purchased from ThermoFisher Scientific (Monza, Italy).

### Preparation of SLN

#### Plain and 6-Coumarin-loaded SLN

Plain SLN were produced according to the melt-emulsion method [14]. To investigate their property of internalization by the cells, these nanoparticles were loaded with 6-Coumarin (6-Coum), a green fluorescent fluorophore. Based on the melt-emulsification method, Gelucire^®^50/13 (60 mg) was melted at 70 °C in a glass vial, and then 6-Coum (6 mg) was added. The surfactant Tween 85 (60 mg) was heated at the same temperature in the presence of 1.37 ml of water and, then, the aqueous mixture was added to the lipidic melted phase at 70 °C, forming an emulsion by homogenization at 0.205 kHz for 2 min operated by an UltraTurrax model T25 apparatus (Janke and Kunkel, Staufen, Germany). The nanosuspension was cooled at room temperature and the resulting SLN were collected by centrifugation (16,000×*g*, 45 min). All procedures involving 6-Coum were carried out under light protection.

#### GSE containing SLN

In order to obtain SLN loaded with proanthocyanidins-containing GSE, the melt-emulsification method was followed with a slight modification in comparison to 6-Coum loaded SLN. At the beginning, 60 mg of the lipid Gelucire^®^ 50/13 were melted at 80 °C. In a separate vial, 1.37 ml of water containing the surfactant (Tween 85, 60 mg), 6 mg of GSE were incubated up to 30 min at 80 °C to enhance dispersion of the active principle in the surfactant. Additionally, the aqueous phase was pre-treated by homogenization at 0.205 kHz for 2 min with an UltraTurrax model T25 apparatus Then, the resulting mixture in aqueous phase was added to the melted lipid phase at 80 °C in order to obtain an emulsion by homogenization upon the same conditions above described. Finally, the nanosuspension was cooled at room temperature and the resulting SLN centrifuged (16,000×*g*, 45 min, Eppendorf 5415D, Hamburg, Germany).

#### DiR loaded SLN

SLN containing DiR (SLN-DiR) were formulated as follows. The aqueous phase consisted of 1.37 ml of water and 60 mg of Tween 85 whereas the oily phase was initially provided by 60 mg of Gelucire^®^ 50/13 melted at 80 °C. Once the lipid was melt, then 100 µl of a DIR solution in DMSO (1 mg/ml) were pipetted to the oily phase maintained at 80 °C. The procedure followed in order to form the emulsion up to the final production of SLN was the same described above.

### Physicochemical characterization of SLN

Particle size and polydispersion index (PDI) of all prepared SLN were determined in double distilled water by photon correlation spectroscopy (PCS) using a Zetasizer NanoZS (ZEN 3600, Malvern, UK). The determination of the ζ-potential was performed using laser Doppler anemometry (Zetasizer NanoZS) after dilution in KCl (1 mM, pH 7.0).

### Quantification of GSE and DiR in SLN

To determine the amount of incorporated GSE, SLN-GSE were resuspended in double sterile distilled water and proanthocyanidins were extracted according to Bligh and Dyer’s modified method [15]. Total proanthocyanidins according to Folin–Ciocalteu’s method [16] were determined. The absorbance was measured at 750 nm by means of a spectrophotometer (Bio-Rad, iMark-microplate reader) and compared with a standard curve of proanthocyanidins solution. Total phenolic content was expressed as mg proanthocyanidins per mg SLN-GSE. All measurements were performed in triplicate.

For DiR quantification, the tracer concentrations were determined, as previously reported [17], from a standard curve of DiR in DMSO (concentrations ranging from 2 to 80 ng/ml), using a fluorometer instrument (Varian Cary Eclipse, Mulgrave, Australia, Excitation: 750 nm; Emission 779 nm).

### Stability studies of SLN-GSE

Freshly prepared GSE-loaded SLN were evaluated for their particle stability at two different temperature conditions (4 and 37 °C) [18] by incubating the particles without any stirring. Precisely, at 4 °C solvent was double distilled water and particles were incubated up to 3 months. For stability evaluation at 37 °C, particles exposure was carried out up to 48 h in Simulated Lung Fluid (SLF), prepared according to our previous work [19]. SLN dispersions were mixed with this medium at 0.1%, v/v ratio. Particle size of all the samples was determined at scheduled time intervals by PCS. Each experiment was performed in triplicate.

### Cell cultures

All biological assays were performed by using the airway epithelial cell line H441, obtained from papillary adenocarcinoma of the lung [20]. Cells were grown in RPMI-1640 medium supplemented with 10% FBS, 1% l-glutamine, and 1% penicillin/streptomycin.

### Propidium iodide exclusion assay

To determine whether nanoparticles affect H441 cells viability, after incubation with different doses and for different times, cells were detached and propidium iodide was added to each sample (25 µg/ml), as previously published [21]. After 20 min on ice the tubes were gently mixed before analysis with AMNIS flow cytometer. Brightfield scatter plots obtained by plotting area (a parameter relative to cellular dimension) on x-axis vs aspect ratio (a parameter reflecting the ratio of the cell Minor Axis divided by the Major Axis) on y-axis were generated to identify single cells events, then 10,000 single-cell events for sample were acquired. As positive control, cells were treated with 0.1% Triton X-100 for 5 min and analyzed. Dead cells were estimated as the percentage of the positive fluorescent cells.

### MTT assay

After incubation with different doses of SLN and for different times, MTT assay was performed on H441 cells, as previously published [20]. Briefly, a stock solution of MTT(3-(4,5-dimethyl-thiazol-2-yl)-2,5-diphenyl tetrazolium bromide) in phosphate buffered saline (PBS) (5 mg/ml) was added to each well reaching a final concentration of 0.5 mg/ml (in 1000 ml of complete medium). After 4 h the formazan crystals were dissolved in DMSO and measured spectrophotometrically by an ELISA reader at a wavelength of 570 nm with a reference wavelength of 690 nm. The relative metabolic activity was calculated in respect to untreated cells (considered as 100%). Triton X-100 treated cells were used as positive control.

### Apoptosis and necrosis assay

H441 cells were incubated with different doses of SLN and for different times and evaluated for apoptosis/necrosis rate with AMNIS flow cytometer, as previously published [22]. Briefly, cells were stained with Annexin V conjugated with a sensitive dye CF647 (excitation laser: 642 nm, emission max: 670 nm) for 15 min at 37 °C, washed in assay buffer, then stained with 7-AAD (excitation laser: 488 nm, emission max: 642 nm; 5 min). Brightfield aspect ratio vs brightfield area plots were generated to identify single cells events, then 20,000 single-cell events for sample were acquired. Dot plots were obtained by plotting the fluorescence of Annexin V (channel 11) vs fluorescence of 7-AAD (channel 5), resulting in different populations: (1) healthy cells, Annexin V(−) and 7-AAD(−); (2) necrotic cells, Annexin V(+) and 7-AAD(+); (3) early apoptotic cells, Annexin V(+) and 7-AAD(−); (4) late apoptotic cells, Annexin V(−) and 7-AAD(+).

### Internalization assay

To evaluate the efficiency of internalization of SLN by airway epithelial cells, H441 cells were incubated with SLN carrying 6-Coum at the final concentration of 0.2, 1 and 10 µg/ml. After 4, 8 or 24 h of incubation with SLN, cells were treated or not treated with trypan blue (0.05% in PBS) to quench extracellular fluorescence [20]. Then cells were detached, resuspended in 50 µl PBS and then analyzed by Amnis Flowsight IS100. Brightfield scatter plots obtained by plotting area on x-axis vs aspect ratio on y-axis were generated, then single cells events were gated, and finally 20,000 single-cell events for sample were acquired. The percentage of green positive cells (channel 2, 488 nm excitation laser) and mean fluorescence were analyzed using Amnis IDEAS software subtracting the values of the negative control. Brightfield and green fluorescent images for any single cell event were collected.

In another set of experiment, H441 cells were incubated with SLN carrying 6-Coum at the concentration of 0.2, 1 and 10 µg/ml for 4 h. Then medium was removed, cells were washed with PBS and fresh medium was added. Cells were split each every 3 days in order to analyse them up to 17 days. Cells were analyzed by Amnis Flowsight IS100 at different days of incubation, in presence and in absence of trypan blue treatment.

### In vivo bioimaging

The distribution of SLN in C57Bl/6 male mice lungs was investigated with DiR as near-infrared fluorescent probe. Six-seven week old male C57Bl/6 (26–28 g) mice were anesthetized by an intraperitoneal injection of a solution of 2.5% Avertin (2,2,2-tribromoethanol and tert-amyl alcohol) (Sigma-Aldrich, Italy) in 0.9% NaCl and administered in a volume of 0.015 ml/g of body weight. Mice were suspended at a 45° angle by the upper teeth. SLN-DiR were administered directly via spray instillation to the lungs using a MicroSprayer aerosoliser (IA-1C; Penn-Century, Philadelphia, PA, USA) suitable for mice, attached to a high-pressure syringe (FMJ-250; Penn-Century). The light source’s (lamp type FLQ85E; Helmut Hund, Germany) flexible fiber-optics arm was adjusted to provide optimal illumination of the trachea. A small spatula was used to open the lower jaw of the mouse and blunted forceps were used to help displace the tongue for maximal oropharyngeal exposure. After a clear view of the trachea was obtained, the MicroSprayer tip was endotracheally inserted and 150 µl of suspension was sprayed. The tip was immediately withdrawn and the mouse was taken off the support. SLN suspension was administered so as to obtain a DiR-dosage of 30 µg/25 g of body weight. At 1, 2, 3, and 6 days (n = 2 for each time point) after drug administration, mice were anesthesised and the SLN-DiR distribution in lungs assessed using a small animal imaging system (IN VIVO F-PRO, Bruker, Germany).

### Evaluation of anti-oxidant activity

To evaluate the efficiency of anti-oxidant activity, H441 cells were incubated for 24–72 h with SLN-GSE (or free GSE as control) at three different doses (2.5, 5 and 10 µM). Then, in order to induce oxidative stress, cells were treated with H_2_O_2_ (0.1 mM) for 24 h. Reactive oxygen species (ROS) production was evaluated by fluorimetry by using the 2′,7′-dichloro-dihydro-fluorescein diacetate (H_2_DCFDA), as previously described [23] and flow-cytometry. The dose and timing of H_2_O_2_ incubation with cells was chosen based on preliminary experiments that evaluated significant ROS production in the absence of overt reduced viability, as assessed by the MTT method.

### Translocation of the nuclear transcription factor NF-κB

H441 cells grown on 96 well plate were prior incubated with SLN-GSE or free GSE for 24, 48 and 72 h, then cells were stimulated with H_2_O_2_ (0.2 mM) for 3 h before evaluation of nuclear translocation of the activated p65 subunit of NF-κB. Cells were fixed in 3% paraformaldehyde and 2% sucrose in PBS and permeabilised with ice-cold Triton HEPES buffer (20 mM HEPES, 300 mM sucrose, 50 mM NaCl, 3 mM MgCl_2_, 0.5% Triton X-100) for 5 min at room temperature. Cells were incubated with blocking solution (2% BSA and 2% FBS), for 15 min at 37 °C, and then with Alexa Fluor^®^ 488-conjugated anti-NF-κB p65 antibody (clone E379, Abcam, Cambridge, MA, USA) for 24 h at 4 °C. Then cells were washed three times with PBS and nuclei were counterstained with 4′,6-diamidino-2-phenylindole (DAPI). Fluorescent images were collected using ZOE™ Fluorescent Cell Imager (Bio-Rad, Segrate, Italy). In order to quantify nuclear translocation of NF-κB, images were analyzed with ImageJ v. 1.51 software (https://imagej.nih.gov/ij/) as previously described [24]. Briefly, three fields were selected for analysis of each stain. The DAPI staining mask was used to define the nuclear region of interest (ROI). Using the image calculator, the threshold DAPI mask was subtracted from threshold NF-κB image to create a cytoplasmic ROI. Nuclear and cytoplasmic staining intensities for each field were used to calculate nuclear:cytoplasmic ratio as relative measure of NF-κB nuclear translocation.

### Statistical analysis

Statistical significance of differences was evaluated by a two-tailed unpaired Student’s *t* test or ANOVA with Tukey’s Multiple Comparison test. Data were analysed by using Prism 5 (Graph-Pad Software, Inc., La Jolla, CA, USA). Differences were considered significant at 95% level of confidence (p < 0.05).

## Results

### Biocompatibility of SLN

In order to evaluate the biosafety of SLN towards airway epithelial cells, different methods were used, including propidium iodide exclusion assay, MTT assay, and necrosis/apoptosis assay, that investigate various aspects of cell viability.

**Fig. 1:**
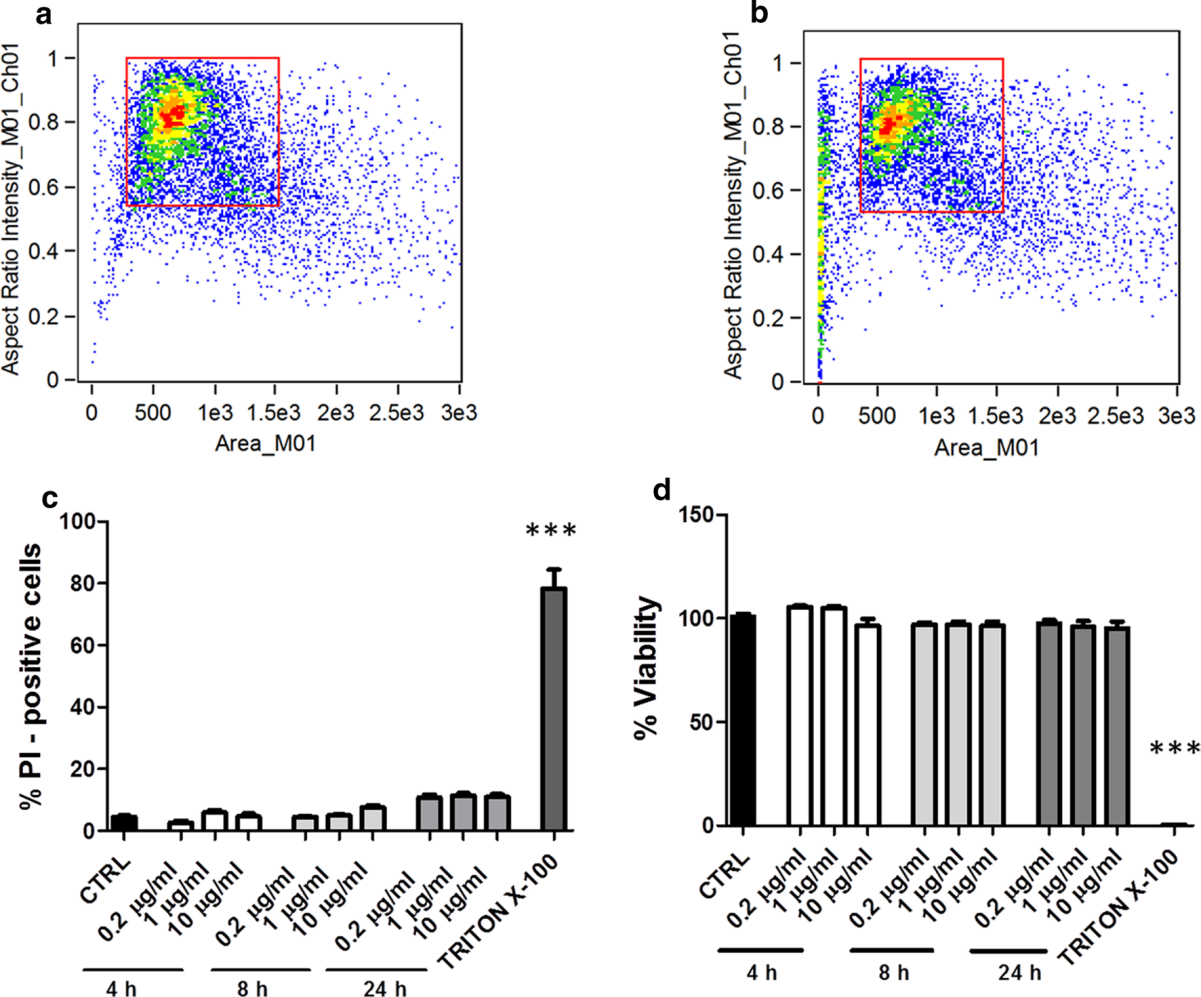
Cytotoxicity of SLN. After incubation with three different doses of SLN and for different times, cells were stained with propidium iodide and analyzed by flow cytometry. Representative dot plots, obtained by plotting the area of the cells (x-axis) vs the aspect ratio (i.e. the ratio between length and height) parameter (y-axis), and the gated population (in red square) are shown for (**a**) untreated cells (CTRL) and (**b**) in presence of treatment with Triton X-100. **c** Percentages of positive cells (PI-positive cells) for the different conditions are shown. The treatment with Triton X-100 (positive control) caused the 80% of cell death. Data are represented as a mean ± standard deviation of three experiments conducted each in duplicate. ***p < 0.001 for Triton X-100 vs CTRL. **d** Cells were evaluated for the viability by the MTT assay. Control (CTRL) is represented by untreated cells, while positive control is represented by cells treated with Triton X-100. Data are shown as a mean ± standard deviation of three experiments conducted each in duplicate. ***p < 0.001 for Triton X-100 vs CTRL

Propidium iodide (PI) is a fluorescent molecule that penetrates only in non-viable cells with alterations of the cell membrane and its internalization within the cells can be detected by flow cytometry. Therefore, the percentage of positive cells for PI is a parameter that reflects cytotoxicity. Dot plot analysis showed a higher presence of cell debris in Triton X-100-treated cells (Fig. 1a, b). However, the treatment with Triton X-100 caused a significant cell death (78.6 ± 6.0%), as indicated by the percentage of PI-positive cells (Fig. 1c). A low and non-significant toxicity of nanoparticles at all three doses and after 4, 8 and 24 h of incubation was observed. A slight but not significant increase of fluorescent signal was noted after 24 h (10.83 ± 1.3% at 0.2 μg/ml; 11.47 ± 1.0% at 1 μg/ml and 10.96 ± 1.4% at 10 μg/ml).

MTT test is a measure of mitochondrial dehydrogenase activity and an evaluation of the cell vitality. High absorbance values reflect appropriate levels of cell viability. This assay confirmed the low cytotoxicity of nanoparticles with a slight but not significant decrease in cell metabolic activity at the highest dose for all time considered (Fig. 1d). Again, only Triton X-100 determined a significant reduction in the metabolic activity.

**Fig. 2:**
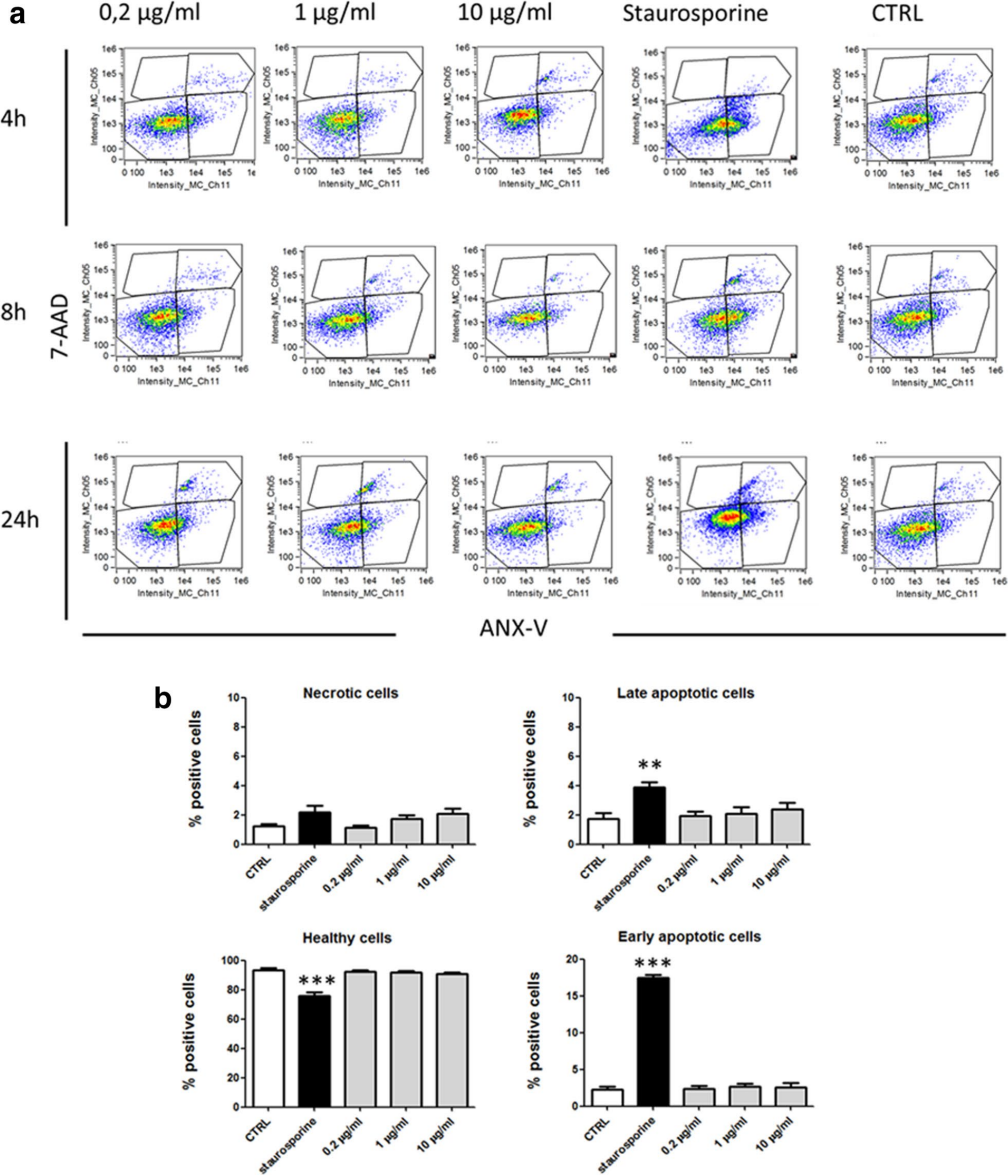
Effect of SLN on necrosis/apoptosis. H441 cells were analyzed for necrosis/apoptosis levels after incubation with different doses of SLN for 4, 8 and 24 h. As positive control cells were treated with staurosporine (1 µM). **a** Representative Annexin V vs 7-AAD dot plots are shown for all time points and SLN doses. **b** Histograms of the results obtained at 24 h are shown. Data are shown as a mean ± standard deviation of three experiments conducted each in duplicate. **p < 0.01; ***p < 0.001

A further biosafety analysis of nanoparticles was conducted by evaluating a possible necrotic/apoptotic effect on the cells. After an incubation for 4, 8 and 24 h with the three doses of nanoparticles used above, the cells were labeled with the antibody anti-annexin V and with the 7-AAD dye that penetrates the non-viable cells and then analyzed by flow cytometry. The results showed the absence of a significant necrotic or pro-apoptotic effect when compared to untreated controls at all the time considered. Dot plots for all experimental conditions are reported in Fig. 2a, while Fig. 2b shows the results obtained only after 24 h of incubation, the results obtained at 4 and 8 h being very similar. In the assay, a positive apoptosis control was obtained by treating the cells with the staurosporine, an apoptosis inducer [25] which caused a significant decrease in viable cells and an increase in percentage of necrotic/late apoptotic cells.

### Internalization efficiency and persistence of intracellular SLN

**Fig. 3:**
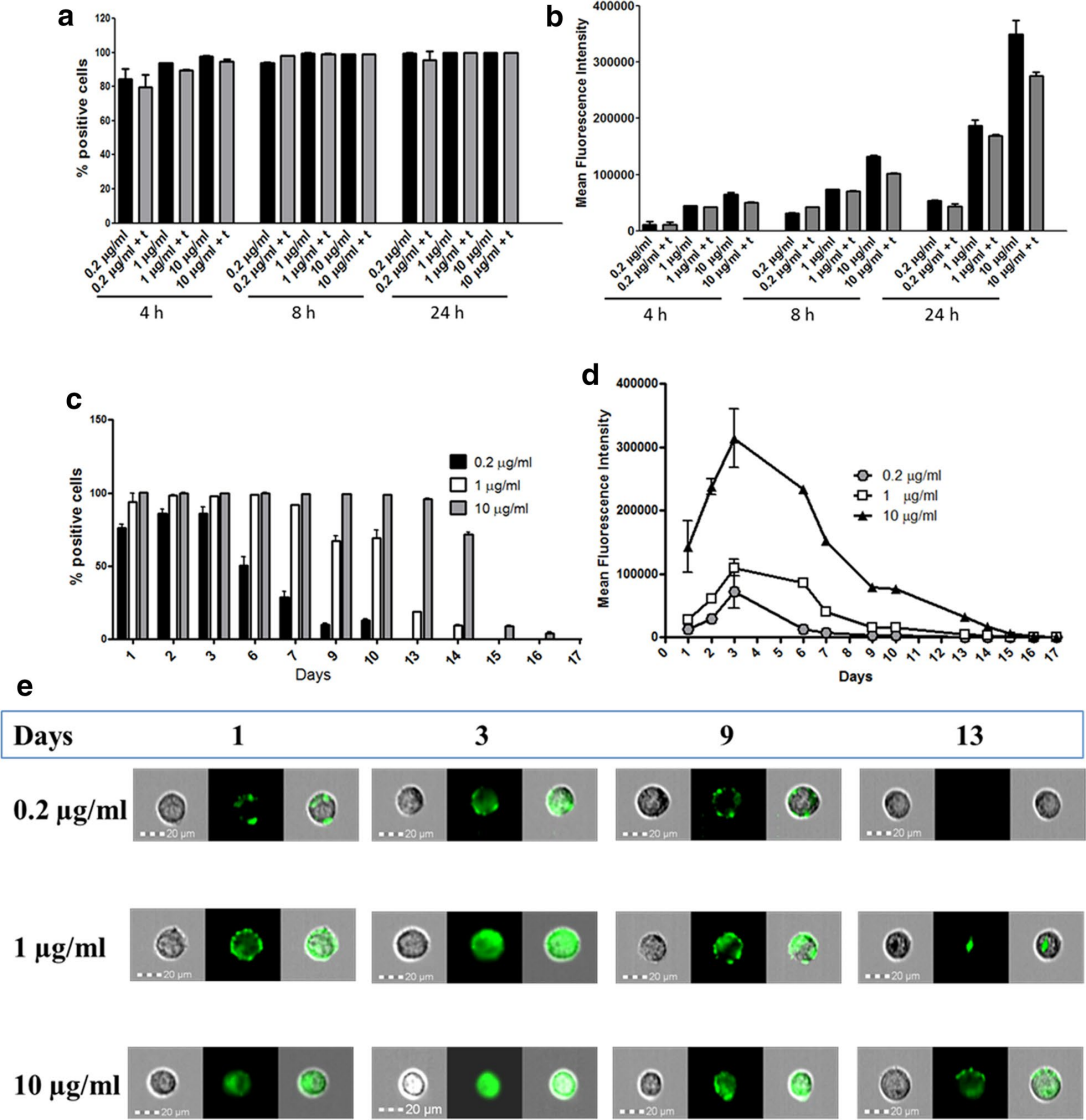
Efficiency of SLN internalization and its time course. Cells were incubated with fluorescent SLN at the times and doses indicated and then percentage of fluorescent cells (**a**) and the mean fluorescence (**b**) was measured by flow cytometry. Black columns indicate samples without trypan blue treatment, while grey columns indicate samples treated with trypan blue. Data are shown as mean ± standard deviation of three experiments conducted each in duplicate. Cells were incubated for 4 h with the three doses of SLN and then the percentage of fluorescent cells (**c**) and the mean fluorescence intensity (**d**) were measured by flow cytometry up to 17 days. Data are represented as mean ± standard deviation of three experiments conducted each in duplicate. **e** Brightfield, green fluorescent images and merge of representative single cells at 1, 3, 9 and 13 days are shown

A cell uptake study was performed by flow cytometry in order to demonstrate the capacity of internalization of nanoparticles within the cells. To this aim, SLN were loaded with 6-Coum, a fluorescent molecule, so providing nanocarriers able to perform cell tracking. The percentage of cells positive for the fluorescence was very high at the lowest dose and only after 4 h of incubation (Fig. 3a). As showed by the treatment with trypan blue that quenches the fluorescence associated to cells but not internalized, most nanoparticles were actually penetrated into the cells. Figure 3b shows that the mean fluorescence intensity is high already at earlier times and increase further at 24 h. The persistence of the fluorescence within the cells over time has been considered, since this aspect is crucial for the controlled drug delivery. The cells were incubated with the nanoparticles for 4 h, then nanoparticles non-associated with cells were removed by washing with PBS and finally the percentage of positive cells for 6-Coum fluorescence was evaluated at different time points (Fig. 3c). The percentage of positive fluorescent cells was high (95–100%) at the intermediate dose (1 µg/ml) and at the highest dose (10 µg/ml) after 24 h of treatment and remained high up to the sixth day for the intermediate dose and up to the tenth day for the highest dose. At the lowest dose (0.2 µg/ml), the highest percentage of positive cells (86.1%) was observed at the second and third day after incubation. For all doses taken into account, the percentage of positive cells decreased gradually and slowly turning into absence of positive cells after 17 days (at 10 μg/ml). Similarly, it is possible to observe an average fluorescence peak after 3 days of treatment for all doses taken into account. This fluorescence decreased gradually at each dose considered (Fig. 3d, e).

In studies involving pulmonary drug delivery by nanoparticles, it is fundamental to assess whether the dosage has been effectively administered into the lungs. Real time in vivo fluorescence imaging is a very efficient technology for studying nanoparticles deposition in tissues. Temporal and spatial data can be collected by labeling drugs with a fluorescent molecule such as a dye, prior to administration into an animal model [26]. In the present study, DiR, a near infrared (NIR) fluorescent cyanine dye that emits in the 600–900 nm range, was chosen for its lipophilic nature that allows it to be incorporated in cell membranes and for its emission that avoids tissue autofluorescence [27].

After intratracheal administration of DiR-loaded SLN using the spray instillator, at different time points (1, 2, 3 and 6 days) mouse thorax was analysed by in vivo fluorescence imaging system. Serial images of the lungs showed persistent disposition of DiR-loaded SLN through the tracheobronchial tree (Additional file 1: Fig. S1). Of important note, DiR-loaded SLNs were observed in mice lungs even at 6 days after drug administration, suggesting that SLNs could remain in the lungs and release DiR in a sustained way.

### Characterization and stability of GSE-loaded SLN

GSE-loaded SLN were characterized for their size, PDI, zeta potential and content of proanthocyanidins (Table 1). From PCS, GSE SLN exhibited mean diameter values and PDI range equal to 243 nm and 0.41–0.51, respectively and, overall, were bigger than the corresponding control particles (141 ± 11 nm [14]; p < 0.001).

**Table 1:** Main physicochemical properties of GSE-loaded SLN, DiR loaded SLN, and 6-Coum-loaded SLN

Formulation	Size (nm)	PDI	Zeta potential (mV)	Content of dye/drug in SLN (mg/mg)
SLN-GSE	243 ± 24	0.41–0.51	− 14.5 ± 1.0	0.058 ± 0.003
SLN-DiR	98 ± 2	0.27–0.41	− 16.9 ± 0.4	0.014 ± 6 × 10^−4^
SLN-6-Coum^a^	235 ± 14	0.51–0.58	− 16.3 ± 1.9	0.41 ± 0.01

Data are mean ± SD of six formulation replicates

^a^From Ref. [14]

**Fig. 4:**
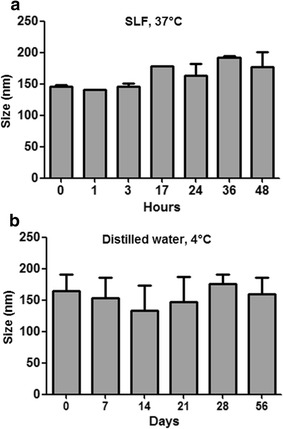
Size stability of SLN-GSE. **a** SLN-GSE were incubated up to 48 h in Simulated Lung Fluid (SLF) at 37 °C. **b** SLN-GSE were incubated up to 2 months in double distilled water at 4 °C. Values represent mean ± SD (n = 3 for each temperature setting). No statistically significant differences were found among time points in both **a**, **b**

Concerning zeta potential values, negatively charged particles were obtained, irrespectively of GSE presence. Moreover, GSE-loaded SLN were evaluated for their size stability at 37 °C in SLF up to 48 h (Fig. 4a) and at 4 °C in double distilled water up to 2 months (Fig. 4b) by measuring their size. These nanoparticles were found capable to retain their mean diameter in both conditions.

The content of proanthocyanidins in SLN was measured up to 60 days and remained constant (Table 2).

**Table 2:** Content of proanthocyanidins in SLN at 4 °C up to 2 months

Time (days)	Proanthocyanidins content in SLN (mg/mg)
0	0.059 ± 0.003
15	0.061 ± 0.004
30	0.060 ± 0.003
40	0.058 ± 0.004
60	0.054 ± 0.007

Data are mean ± SD of six formulation replicates. No statistically significant differences were found among time points

### Efficacy of GSE-loaded SLN on oxidative stress

**Fig. 5:**
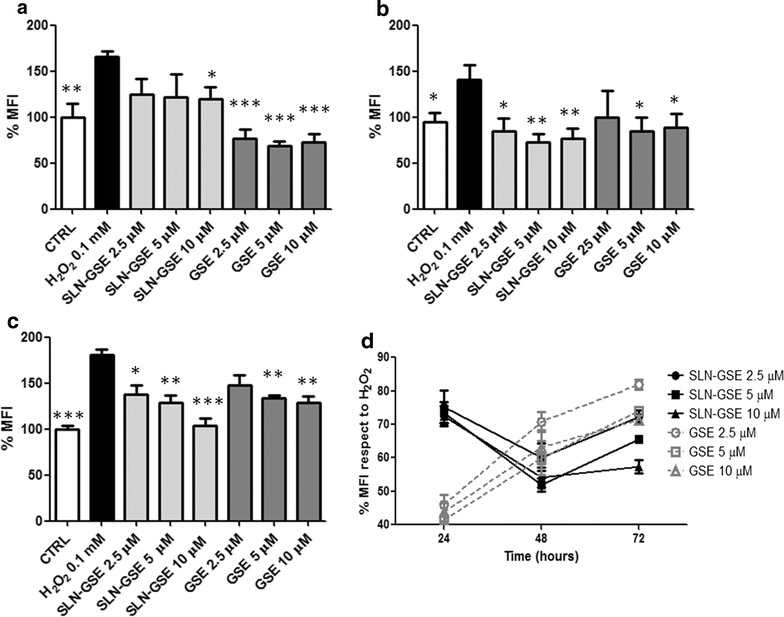
Efficacy of SLN-GSE on oxidative stress. H441 cells were incubated with three different doses of SLN-GSE and free GSE 24 h (**a**) 48 h (**b**) and 72 h (**c**) before treatment with H_2_O_2_, and the relative mean fluorescence (%) was measured by flow-cytometry after incubation with H_2_DCFDA. **d** Time course of % MFI at each time point respect to fluorescence obtained in presence of treatment with H_2_O_2_ (considered as 100%). Data are shown as mean ± standard deviation of three experiments conducted each in duplicate. *p < 0.05 **p < 0.01 ***p < 0.001 for H_2_O_2_ vs all conditions

We next investigated the efficacy of SLN-GSE in terms of reduction of ROS production. First we studied which doses of hydrogen peroxide (H_2_O_2_) induced an oxidative stress in the absence of an overt cellular toxicity. As shown in Additional file 2: Table S1, treatment with 0.1 mM H_2_O_2_ for 24 h caused a high production of ROS with a low cytoxicity as compared with the other conditions, thereby we decided to use 0.1 mM for 24 h. Pre-treatment of H441 cells with three different doses of SLN-GSE (or free GSE) 24 h prior to the oxidative stimulus resulted in a significant reduction of the relative mean fluorescence at the highest dose of SLN-GSE while free GSE was effective at the three doses used (Fig. 5a). To assess if a single administration of SLN-GSE was more effective in comparison with free GSE after longer times, we incubated H441 cells with SLN-GSE and free GSE, 48 and 72 h prior to the oxidative stimulus. Pretreatment of 48 and 72 h resulted in a significant reduction of the mean fluorescence at the three doses of SLN-GSE used, while free GSE was no more effective at the lowest dose used (Fig. 5b, c). Figure 4d shows the variation of the percentage of the mean fluorescence relative to the mean fluorescence detected after treatment of H_2_O_2_ alone at any time point (100%). These results indicate that encapsulation of proanthocyanidins allows them to be more efficacious for longer times than free compound.

### Effect of SLN-GSE on NF-κB nuclear translocation mediated by H_2_O_2_

**Fig. 6:**
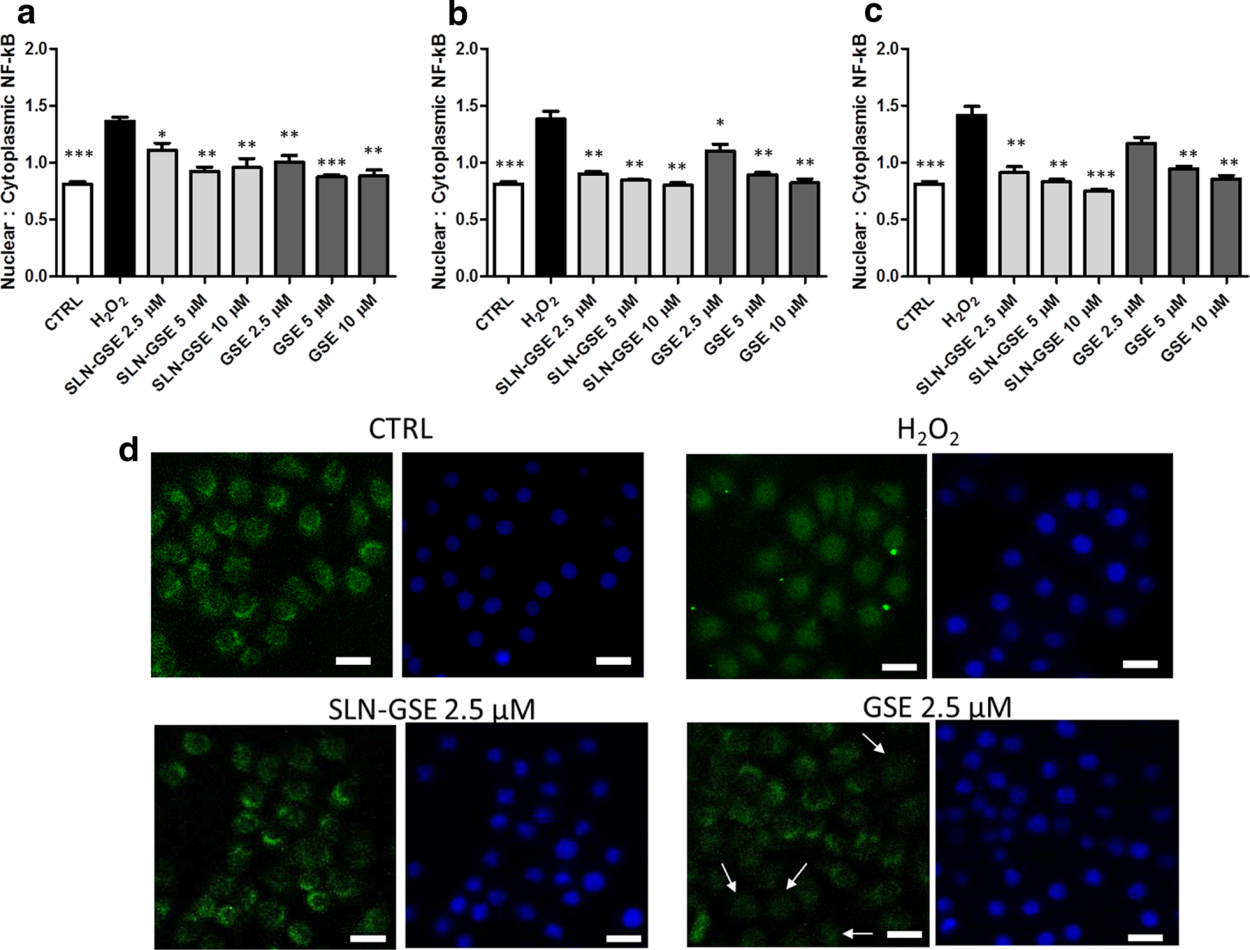
Effect of SLN-GSE on NF-κB nuclear translocation mediated by H_2_O_2_. H441 cells were incubated with three different doses of SLN-GSE and free GSE 24 h (**a**), 48 h (**b**), and 72 h (**c**) before treatment with H_2_O_2_, then immunodetection of NF-κB was performed, and the nuclear:cytoplasmic fluorescence ratio was calculated. Data are  shown as mean ± standard deviation of three experiments conducted each in duplicate. *p < 0.05 **p < 0.01 ***p < 0.001 for H_2_O_2_ vs all conditions. **d** Representative images of NF-κB signal (green) and nuclear signal (blue) obtained with untreated cells (CTRL), treated with H_2_O_2_, or incubated with the lowest doses (2.5 µM) of SLN-GSE and free GSE for 72 h before H_2_O_2_. Cells in the free GSE panel with NF-κB nuclear staining only are indicated by white arrows. Images were obtained with an original magnification of ×20 and after zooming (×4). Scale bar: 20 µm

To study the efficacy of SLN-GSE on downstream pathway(s) of inflammation induced by H_2_O_2_, we investigated the effect of pretreatment with SLN-GSE on nuclear translocation of NF-κB. First, we determined which dose of H_2_O_2_ and time of incubation induced a detectable NF-κB nuclear translocation from cytoplasm in H441 cells, obtaining a significant effect by using 0.2 mM H_2_O_2_ for 3 h (data not shown). Thus, H441 were pretreated for 24, 48 and 72 h with the three doses of SLN-GSE or free GSE, then H_2_O_2_ was added for 3 h before NF-κB immunodetection. Pretreatment with SLN-GSE or free GSE resulted in a significant reduction of nuclear:cytoplasmic ratio with an increased effect by prolonging the time of incubation (Fig. 6a–c). Interestingly, after 72 h of treatment, the lowest dose of free GSE was less effective as compared to previous time points, while the SLN-GSE retained its efficacy (Fig. 6c). In relation to this time point, we show representative images of control cells (CTRL), cells treated with H_2_O_2_ and pretreated either with GSE-SLN or free GSE before H_2_O_2_ (Fig. 6d). In CTRL, NF-κB is cytosolic, whereas H_2_O_2_-treated cells show a clear nuclear staining. While cells pre-treated with GSE-SLN returned to the control cytosolic pattern of staining in almost all cells, free GSE-treated cells showed a limited efficacy (as shown by nuclear stained cells, indicated by the white arrows).

## Discussion

In COPD pathogenesis, cigarette smoke (CS) and/or pollutants are responsible for the three main features: inflammation, protease/antiprotease imbalance and oxidative stress due to high production of ROS [5]. The airway epithelium plays a fundamental role in recognition of inhaled microorganisms (bacteria, viruses, and fungi) by innate immune receptors (such as Toll-like receptors, lectin-type receptors and intracellular receptors) that activates several signaling pathways, leading ultimately to the secretion of antimicrobial peptides and proteins (defensins, cathelecidins, lactoferrin, lysozyme), as wells as cytokines and chemokines [28]. This array of molecules is involved in the recruitment of innate and adaptive immune cells, contributing to and amplifying airway inflammation. CS has multiple effects on airway epithelial cells, including ROS production [29–31], cell proliferation by EGFR activation [32], mucus cell differentiation and mucin production via activation of TACE, release of TGF-α and EGFR phosphorylation [33]. ROS have been implicated in the activation of TACE [33], mucin production [34], and CXCL-8 secretion [35]. CS increases intracellular ROS levels in lung epithelial cells via activation of NAPDPH oxidase [36, 37], upregulating several ROS-sensitive signaling pathways, including the mitogen-activated protein kinases (MAPKs) and various downstream transcriptional factors, such as nuclear factor-κB (NF-κB), and ultimately promoting inflammatory gene expression [36–40].

Among pulmonary drug delivery systems, SLN possess many advantages such as: biocompatibility and biodegradability (when they are composed of well tolerated excipients), can be large-scale produced [41] and, finally, confer stability to the incorporated drugs. Moreover, they display excellent physical stability. Such particles in order to escape macrophage clearance, should be smaller than 260 nm [42], and facilitate deep-lung deposition if they are smaller than 500 nm, thus providing a prolonged residence time in the lungs, and consequently increasing the therapeutic effect of compounds [43–45]. As a result of these properties, SLN have been investigated for the purpose of inhaled drug delivery in the treatment of pulmonary diseases [46] and they could be an appropriate carrier for delivering lipophilic molecules such as procyanidins. These phenolic compounds from the flavonoid groups have been extensively studied in animal models and humans, because they function as potent anti-oxidants, anti-aging and anti-inflammatory agents [47–49]. Moreover, they show cardioprotective [50] and anticancer properties [51].

In this study, we have characterized the shelf stability, biocompatibility and uptake of plain and fluorophore-loaded SLN, respectively, with a cellular model of airway epithelium. As for the low loading capacity observed for the examined SLN, it is comparable with that observed in the encapsulation of proanthocyanidins in a hydrophilic polymeric matrix as chitosan [52] rather than in a hydrophobic one as PLA [53]. This outcome may be accounted for considering the peculiar features of the lipid used in the preparation of GSE-SLN, i.e., Gelucire^®^ 50/13. According to manufacturer instructions, this lipid, indeed, is composed of well-characterized PEG-esters (Stearoyl polyoxyl-32 glycerides), a small glyceride fraction and free PEG and is able to self-emulsify on contact with aqueous media suggesting so a partial hydrophilic character of the lipid Gelucire^®^ 50/13. Nonetheless, drug loading of our SLN (5.8%) is comparable to that of SLN incorporating resveratrol (up to 6.88%) when Phospholipon^®^ 90G was used [54], indicating that this is a general feature independent of surfactant employed. Further studies ought to focus on the improvement on drug loading in SLN. Our formulation of SLN was stable at 4 °C and retained its payload up to 2 months, confirming previously published results by others with stearic acid-containing SLN when refrigerated [55]. SLN were not cytotoxic at all concentrations tested and this comfortable result foresees a safe application of these nanoparticles in vivo. We observed that SLN were rapidly internalized by airway epithelial cells, accumulated with a peak at day 3, and remained within them for a reasonable time to perform their antioxidant and anti-inflammatory activities. A possible explanation of the 6-Coum accumulation could be due to the burst release of encapsulated compounds in vitro from SLN, that occurs in the first hours, and likely attributable to the release of compounds adsorbed on the surface of nanoparticles [56–58]. After an initial burst, whose peak was at day 3, there was a decline in the intracellular fluorescence. There are two possible explanations for the decrease in the 6-Coum-associated signal: the degradation of the fluorescence molecule, or the cell cultures split. Nevertheless, the long presence of intracellular SLN (up to 16 days upon a single administration) should avoid too many repeat doses in vivo, which is fruitful for augmenting their compliance in the chronically affected patient. However, we do not know the mucopenetration capacity of these SLN, an important parameter for in vivo applications. Concerning the size of SLN-GSE greater than SLN-DiR it may be questioned that a different uptake profile between the two particle types examined may occur. In this regard, it should be taken into account that actually the uptake into cells depends strongly on nanoparticle size. However, even further factors such as surface charge, cell type and degree of aggregation play an important role and, at present, it is not possible to draw definitive conclusions about the cellular uptake profile based on a single property as the particle size [59]. The size stability of SLN in SLF [60] at 37 °C up to 48 h suggests that this SLN formulation might deliver proanthocyanidins at appropriate times in a more complex environment such as that occurring in vivo. We studied preliminarily the uptake of SLN loaded with an appropriate fluorochrome, (DiR, whose properties when formulated as SLN are mentioned in Table 1) and in vivo bioimaging in mice lungs upon their aerosolization, finding that SLN localized in the lungs for at least 6 days after administration. An aspect which is worthy of mention is the very poor lung biodistribution of free DiR, in mice. Indeed, Huang et al. [61] have shown that upon intratracheal administration of this NIR-dye, the fluorescence signal in the lung, even after 15 min, is very low. On the contrary, the co-administration of this fluorophore with surfactant, which is mainly composed of lipids, improved the fluorescence signal associated to this dye. Since macrophages play a fundamental role in particle clearance from the lung, further experiments with SLN-DiR have to be designed to understand their role in in vitro and in vivo uptake.

ROS production in H441 cells was induced by H_2_O_2_, which has been previously used to induce oxidative stress in a variety of cell types [62–64]. Because H_2_O_2_ can determine cell death easily permeating through the membranes [62], we evaluated the H_2_O_2_ concentration and timing of exposure of cells to H_2_O_2_ in order to optimize ROS production without affecting viability (0.1 mM for 24 h). Under these experimental conditions, ROS production was significantly affected by both free GSE and SLN-GSE. While free GSE was more effective in reducing ROS levels at 24 h, SLN-GSE showed a higher anti-oxidant activity at longer times. At 24 h, our results were comparable to those achieved by others when simple S-carboxymethylcysteine (S-CMC) or *N*-acetyl-l-cysteine (NAC) was used to counteract ROS production induced by IL-1β in human pulmonary epithelial H292 cells [65], suggesting that proanthocyanidins are as effective as more studied compounds. This result was paralleled by that on NF-κB activation, indicating that this formulation might be efficacious also in vivo to dampen ROS-induced inflammation during the onset and maintenance of chronic lung diseases. Differences between SLN-GSE and free GSE in reduction of both oxidative stress and NF-κB activation was detected at 3 days, however, controlled release should be further examined at longer times to detect differences between free GSE and SLN-GSE. Although other groups have shown that SLN are capable of delivering other natural anti-oxidant compounds [58], to the best of our knowledge our study is the first to show the delivery of proanthocyanidins by SLN to airway epithelial cells and fluorochrome-containing SLN to the lungs in vivo. Only a previous paper showed that GSE, at the concentrations up to 20 µg/ml, was not cytotoxic to pulmonary A549 epithelial cells and reduced mucin transcript and protein expression as well as various signaling pathways involved in inflammation [66].

## Conclusions

The properties of SLN in terms of biocompatibility, internalization, stability and lung deposition make them as a suitable drug delivery system to the lung. Uptake and persistence of 6-Coum-SLN in airway epithelial cells in vitro strongly suggest that SLN may be retained for a prolonged time when administered in vivo, as demonstrated by bioimaging experiments, should they overcome the mucus barrier. Consequently, GSE-loaded SLN exerted their effect as anti-oxidant agents for longer times than free GSE, suggesting the controlled release of their payload. Overall, our results pave the way to the development of novel natural substance-based pharmaceutics aimed at dampening the oxidative stress and inflammation in chronic respiratory diseases.

## Additional files

**Additional file 1: Figure S1.** In vivo bioimaging. Fluorescence imaging of DiR-loaded SLN deposition in mouse lungs at different days after intratracheal administration. Two mice were aerosolized with DiR-loaded SLN (treated), while one mice was aerosolized with saline (Ctrl). The DiR signal is absent in this control mouse at the level of the thorax.

**Additional file 2: Table S1.** Effect of H_2_O_2_ on ROS production and cell viability in function of dose and time of incubation.

## Abbreviations

SLN: solid lipid nanoparticle
COPD: chronic respiratory obstructive disease
CF: cystic fibrosis
GSE: grape seed extract
ROS: reactive oxygen species
PI: propidium iodide
7-AAD: 7-aminoactinomycin D
MTT: 3-(4,5-dimethylthiazol-2-yl)-2,5-diophenyltetrazolium bromide
PCS: photon correlation spectroscopy
H_2_DCFDA: 2′,7′-dichloro-dihydro-fluorescein diacetate
CS: cigarette smoke
DiR: 1,1′-dioctadecyl-3,3,3′,3′-tetramethylindotricarbocyanine iodide
EGFR: epidermal growth factor receptor
TACE: tumor necrosis factor-α converting enzyme
TGF: transforming growth factor
NADPH: nicotinamide adenine dinucleotide phosphate
PDI: polydispersion index
