# Supplementary figures and images for: Nanoparticle delivery of grape seed-derived proanthocyanidins to airway epithelial cells dampens oxidative stress and inflammation
Source: J Transl Med. 2018 May 23;16:140. doi: 10.1186/s12967-018-1509-4 (PMC5966913; doi:10.1186/s12967-018-1509-4)

Ctrl

Treated

day 1

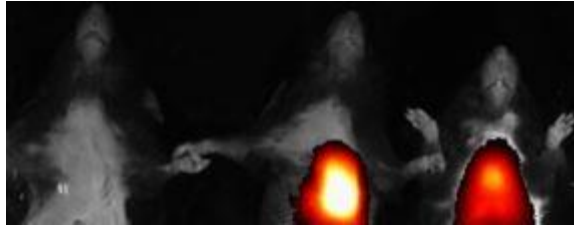

day 2

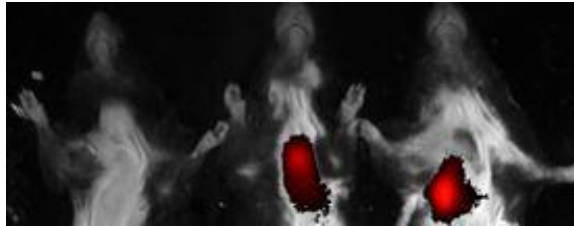

day 3

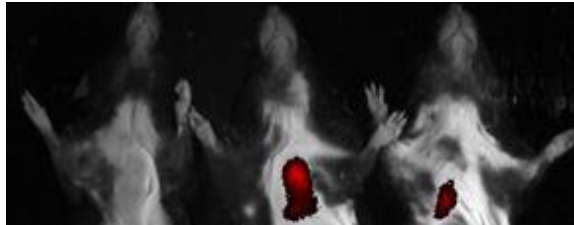

day 6

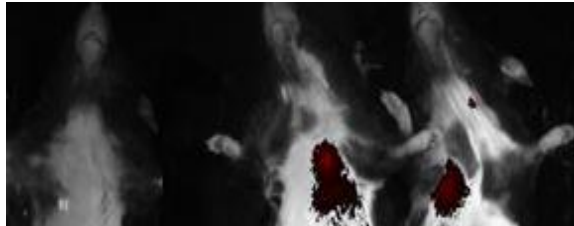

Supplement: Supplementary file 1 — Additional file 1: Figure S1. In vivo bioimaging. Fluorescence imaging of DiR-loaded SLN deposition in mouse lungs at different days after intratracheal administration. Two mice were aerosolized with DiR-loaded SLN (treated), while one mice was aerosolized with saline (Ctrl). The DiR signal is absent in this control mouse at the level of the thorax. [file 12967_2018_1509_MOESM1_ESM.pdf]
