# Supplementary material for: Nanoparticle delivery of grape seed-derived proanthocyanidins to airway epithelial cells dampens oxidative stress and inflammation
Source: J Transl Med. 2018 May 23;16:140. doi: 10.1186/s12967-018-1509-4 (PMC5966913; doi:10.1186/s12967-018-1509-4)
Supplement: Supplementary file 2 — Additional file 2: Table S1. Effect of H2O2 on ROS production and cell viability in function of dose and time of incubation. [file 12967_2018_1509_MOESM2_ESM.docx]

| **Additional file 2: Table S1**. Effect of different doses and times of incubation with H_2_O_2_ on ROS production and viability. | | | | | | |
| --- | --- | --- | --- | --- | --- | --- |
| **Dose and time of incubation with H_2_O_2_** | **0.1 mM/4 h** | **0.5 mM/4 h** | **1 mM/4 h** | **0.1 mM/24 h** | **0.5 mM/24 h** | **1 mM/24 h** |
| % of ROS production respect to CTRL (100%) | 110.0 ± 2.1  (n.s.) | 189.0 ± 28.7 | 376.5 ± 29.4 | 164 ± 28.0 | 739.6 ± 17.3 | 935.6 ± 50.9 |
| % of viability respect to CTRL (100%) | 99.5 ± 0.8  (n.s.) | 29.5 ± 7.8 | 27.8 ± 4.8 | 95.2 ± 1.2  (n.s.) | 8.8 ± 5.9 | 3.1 ± 0.4 |

Data are shown as mean ± SD. CTRL: control.
